# Isolation and Identification of Bacteria from Three Geothermal Sites of the Atacama Desert and Their Plant-Beneficial Characteristics

**DOI:** 10.3390/microorganisms11112635

**Published:** 2023-10-26

**Authors:** Patricio Muñoz-Torres, Sebastián L. Márquez, Germán Sepúlveda-Chavera, Steffany Cárdenas-Ninasivincha, Mabel Arismendi-Macuer, Wilson Huanca-Mamani, Yola Aguilar, Antonio Quezada, Franco Bugueño

**Affiliations:** 1 Laboratorio de Patología Vegetal y Bioproductos, Facultad de Ciencias Agronómicas, Universidad de Tarapacá, Av. General Velásquez 1775, Arica 1000000, Chile; gsepulve@uta.cl (G.S.-C.); sfcninasivincha@gmail.com (S.C.-N.); arismendimabel@gmail.com (M.A.-M.); whuanca@uta.cl (W.H.-M.); catacorayol35@gmail.com (Y.A.); quezadaortega@gmail.com (A.Q.); franco.bugueno.guerrero@gmail.com (F.B.); 2 Fundación Científica y Cultural Biociencia, José Domingo Cañas, 2280 Ñuñoa, Santiago 7750132, Chile; sebastian.marquez@usach.cl

**Keywords:** extreme environments, agriculture, plant growth-promoting bacteria, biocontrol

## Abstract

The Region of Arica and Parinacota (Atacama Desert) offers several unexplored remote sites with unique characteristics that would allow for the formulation of new bioproducts for agriculture. Among them, Jurasi Hot Springs, Polloquere Hot Springs, and Amuyo Lagoons represent a group of open pools fed by thermal water springing from the mountains. Their microbiomes remain unspecified, providing a unique opportunity to characterize the endemic community of these sites and develop new bioproducts for sustainable agriculture. Bacteria were isolated from the sediments of these geothermal sites and characterized by sequencing the 16S rRNA gene, microbiological characterization, and agricultural functional characterization. A total of 57 bacteria were isolated from three geothermal sites north of the Atacama Desert. The sequence analysis showed that the isolates belong to several bacterial genera, including *Pantoea*, *Bacillus*, and *Pseudomonas*, among others. The functional characterization revealed the presence of PGP traits, hydrolytic enzymes, and biocontrol activity against phytopathogenic fungi. These bacteria possess the potential to develop new biobased products for agriculture in arid conditions.

## 1. Introduction

Agriculture faces the challenge of providing safe and nutritious food to a population that will grow from 7.5 billion people today to ~10 billion by 2050 [1]. In addition, inappropriate agricultural practices, natural conditions, poor soil management, and high levels of agrochemicals have reduced the arable land surface, with an increased salinization process and contamination of water and ecosystems [2]. In this sense, it is necessary to adopt practices that allow for, in the long term, maintaining the productivity, viability, and quality of the soil, preserving the environment and its resources so that future generations can use such for the cultivation of plant-based food.

Soil is a critical and necessary resource that helps to maintain any ecosystem and must be adequately and effectively managed to produce healthy crops. Soils are composed of complex microbial communities determined by the environment’s physical and chemical composition. The microorganisms comprising these communities are recognized as a vital component of the world’s biodiversity. They are involved in many activities, such as nutrient recycling, having beneficial relationships with other organisms, contributing to the production of gaseous oxygen, participating in animal and plant health, and playing a critical role in soil fertility, among other processes [3].

From an agricultural point of view, microorganisms are involved in a variety of processes, such as nitrogen fixation (a process that allows elemental nitrogen to be captured and incorporated into biological systems) [4], favoring the solubilization of phosphorus [5] and iron solubilization [6] (elements that are not available to plants and that must be processed by microorganisms prior to their incorporation); the generation of phytohormones that regulate the plant growth [7]; the production of substances with an antagonistic effect against plant pathogens [8]; the induction of plant resistance to pathogens [9]; and increased tolerance to abiotic stress [7]. These microorganisms play a key role in formulating new agricultural bioproducts, which have shown promising results when applied as biostimulants and biocontrol agents [8].

According to Business Wire, the agricultural bioproducts market is expected to grow at a compound annual growth rate of ~11% from 2021 to 2026, reaching a market size of approximately 19,700 million USD in 2026 [10]. However, under this promising scenario, the applications of commercial bioproducts are limited to the sites where the microorganisms were originally obtained or sites with similar environmental conditions and characteristics. For example, commercial strains of the fungus *Trichoderma* show inconsistent results when applied as soil biocontrol agents to prevent infectious diseases in northern Chile, where salinity and the presence of boron affect the growth and sporulation of the fungus, reducing its usefulness as a biocontrol agent [11]. In Chile, for example, the Azapa and Lluta valleys, located in the Arica and Parinacota Region (Atacama Desert), provide the country with vegetables during the winter season, and commercial biocontrol agents have limited activity due to the prevailing saline-boric conditions of this site. From this point of view, it is possible to observe a lack of commercial bioproducts for environments considered extreme sites. Therefore, a strategy to find new agricultural bioproducts could consist of the bioprospection of extreme environments, whose native microorganisms are naturally adapted to the prevailing extreme conditions and could be used as novel biofertilizers, biostimulants, and biocontrol agents.

Extreme environments are habitats characterized by harsh environmental conditions beyond the optimal range for human development. Extreme environments include acidic or alkaline pH, high or low temperatures, saturating salt concentrations, high radiation, and high pressures. Microorganisms from extreme environments are gaining more attention due to their biodiversity, evolutionary traits, and biotechnological applications. In addition, extreme environments are characterized by the seasonal variation in extreme conditions, which allows the development of extremophilic and extreme-tolerant microorganisms [12]. Some species of extremotolerant microorganisms have been described for their ability to act as plant growth promoters, increasing the tolerance to salinity in crops of agricultural interest [13]. Gaete et al. [14] isolated 71 bacterial strains from the Coppermine Peninsula (Antarctica) and Lejia Lake (Atacama Desert); these isolates exhibited in vitro plant growth-promoting properties that could allow for developing new biobased products to mitigate cold or drought stresses. Moreover, Tuesta-Popolizio et al. [15] isolated, identified, and characterized 15 new microbial strains at the Los Negritos geothermal site in Mexico. These strains exhibited activities promoting plant growth, such as auxin production, inorganic phosphate solubilization, and siderophore production, suggesting that geothermal sites could be explored for bacteria enhancing plant growth.

**Figure 1:**
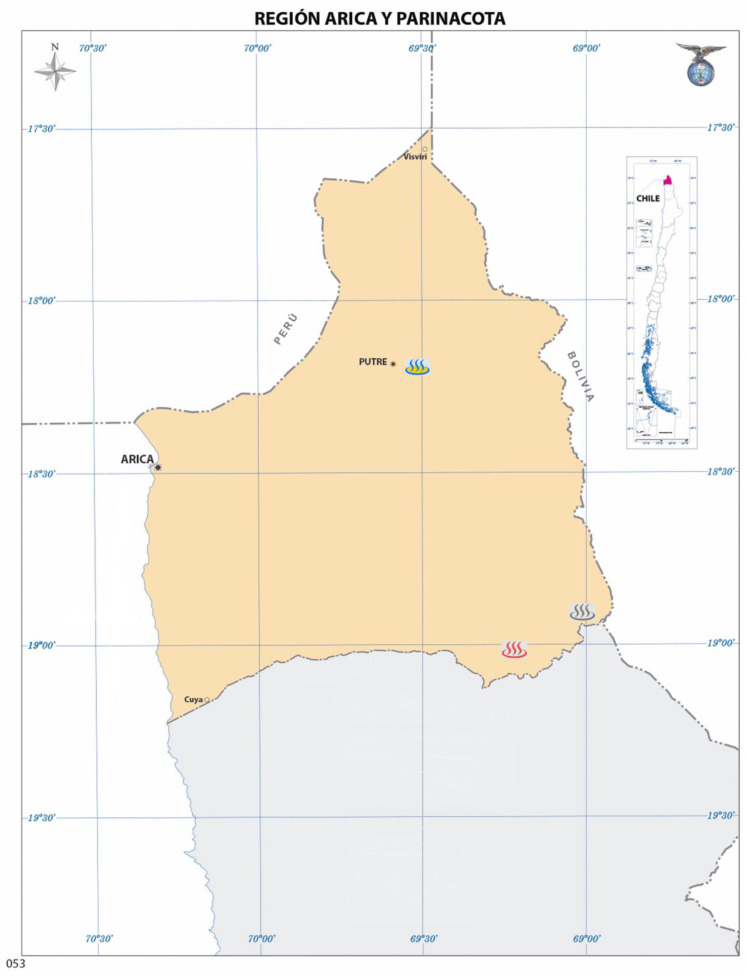
Arica and Parinacota Region (Chile) map. Jurasi Hot Springs, Polloquere Hot Springs, and Amuyo Lagoons are indicated on the map as blue, gray, and red symbols, respectively. This map was obtained and modified from the Chilean Instituto Geográfico Militar website (https://www.igm.cl/ Accessed on 1 March 2022).

The Arica and Parinacota Region has several unexplored extreme sites with unique characteristics that would allow for the formulation of new bioproducts for agriculture. Among these sites, Jurasi Hot Springs, Polloquere Hot Springs, and Amuyo Lagoons (Figure 1) are located in the Andes Mountains and represent a group of open pools fed by a hot water spring emerging from the mountains. The microbiomes associated with these sites have not been previously studied, providing a unique opportunity to characterize the sites’ endemic communities and to develop new bioproducts for sustainable agriculture and biotechnology.

This study focused on the isolation, identification, and characterization of culturable bacteria with the potential for developing agricultural bioproducts, associated with three geothermal sites located in the Arica and Parinacota Region. These bacteria were characterized based on their in vitro plant growth promotion and biocontrol properties.

## 2. Materials and Methods

### 2.1. General

Reagents were purchased from Sigma-Aldrich (St. Louis, MO, USA) or Merck Co. (Darmstadt, Germany). Potato dextrose agar (PDA) was purchased from (Oxoid, ThermoFisher Scientific, Waltham, MA, USA).

### 2.2. Sampling and Bacterial Isolation

Sampling was conducted at Jurasi Hot Springs (Figure 1) in December 2020. Polloquere Hot Springs and Amuyo Lagoons sampling was performed in March and April 2021, respectively.

Sediment samples were aseptically collected using a metallic shovel disinfected with 70% ethanol and stored in sterilized plastic Whirl-Pak^®^ bags (capacity: 532 mL) (Merck, Germany). Water samples were collected by direct immersion of clean 0.5 L plastic bottles. Samples were kept in a cooler at 4 °C and immediately transported to the laboratory for further processing.

Sediment and water samples were analyzed for chemical composition as an external service (Laboratorio de Suelos y Agua, Facultad de Ciencias Agronómicas, Universidad de Tarapacá, Arica, Chile). Analyses were performed using the manual “Métodos de análisis recomendados para los suelos de Chile” (https://biblioteca.inia.cl/handle/20.500.14001/8541 Accessed on 23 October 2023). Bicarbonate ions were determined by the acid-base volumetric method using 0.01 M H_2_SO_4_. The concentration of boron was determined by the azomethine colorimetric method. Calcium, magnesium, sodium and potassium were determined using atomic absorption spectrometry (Agilent, 200 Series AA, Santa Clara, CA, USA). Chloride was determined by the Mohr method. Nitrates were determined using a colorimetric method and measurement at 420 nm. Phosphates concentration was determined by the formation of an ammonium vanadomolybdate complex. Cu, Fe, Mn, and Zn were determined by the DTPA method. As was determined using a colorimetric method based on the used test strips (Merck, Germany).

Environmental sediment samples were directly inoculated into 25 mL of three culture media. King’s B medium, containing 20.0 g peptone, 10.0 mL glycerol, 1.5 g K_2_HPO_4_, and 1.5 g MgSO_4_·7H_2_O per liter. Nutrient broth, containing 3.0 g beef extract and 5.0 g peptone per liter. Modified mineral medium, containing 2.0 g tryptone, 1.2 g NH_4_Cl, 14.0 g NaCl, 2.0 g MgSO_4_·7H_2_O, 0.35 g KCl, 0.3 g CaCl_2_, 0.3 g KH_2_PO_4_, 0.03 g H_3_BO_3_, 0.02 g KI, 0.06 g sodium citrate, 5.0 mL trace solution, and 1.0 mL vitamins solution per liter. Trace solution containing 0.5 g MnSO_4_, 0.1 g FeSO_4_, 0.1 g CoCl_2_, 0.1 g ZnSO_4_, 0.05 g NiSO_4_, 0.01 g CuSO_4_, 0.01 g Na_2_MoO_4_, and 0.001 g NaSeO_3_ per liter. Vitamin solution containing 100 mg/mL biotin and 100 mg/mL pyridoxal·HCl. The pH was adjusted to 7.0. All cultures were incubated aerobically at 25 °C for one week or until microbial growth was observed. Bacteria were isolated from enriched cultures by serial dilutions on liquid medium and streaking on plates of solid medium supplemented with 1.5% (*w*/*v*) of agar. Colonies were transferred to the appropriate liquid medium. Incubations were performed at 25 °C. These procedures were repeated until a single and homogeneous morphology was observed.

### 2.3. DNA Isolation, Amplification, and Sequencing of 16S rRNA Gene

Genomic DNA was isolated using the method described by Huanca-Mamani et al. [16] with modifications. Briefly, bacterial cultures were centrifuged at 5000 rpm for 10 min. The supernatant was discarded, and the bacterial pellet was mixed with 300 μL lysis buffer (2% CTAB, 1.4 M NaCl, 1% PVP, 20 mM Na_2_EDTA, 0.2% LiCl, and 100 mM Tris-HCl pH 8.0), vortexed to homogenize and incubated at 65 °C for 30 min. The mixture was centrifuged at 12,000 rpm for 5 min at room temperature. The supernatant was transferred to a new 1.5 mL centrifuge tube, and an equal volume of chloroform-isoamyl alcohol (24:1 *v*/*v*) was added. The samples were centrifuged at 12,000 rpm for 10 min, and the aqueous phase was transferred to new tubes, adding one volume of 3 M NaCl and 25 μL silica matrix (1 mg/mL), mixed by immersion, incubated at room temperature for 2 min, and centrifuged at 12,000 rpm for 12 s. The pellet was washed twice with 500 μL of a solution containing 50% ethanol, 10 mM Tris-HCl pH 7.5, 100 mM NaCl, and 1 mM Na_2_EDTA. Genomic DNA was eluted by resuspending the silica matrix pellet with 35 μL distilled deionized H_2_O and incubating at 65 °C for 5 min. Samples were centrifuged at 12,000 rpm for 2 min, and the DNA supernatant was transferred to new centrifuge tubes. Genomic DNA was observed on a 1.0% agarose gel prepared in 1X TAE buffer (40 mM Tris-acetate and 10 mM EDTA) and visualized under UV light using 1X GelRed (Biotium, Fremont, CA, USA) prepared in TAE buffer.

The 16S rRNA gene was amplified by PCR using the bacteria-specific primers 27F and 1492R [17]. The reaction mix contained 1.0 U Taq DNA polymerase, 200 μM of each deoxynucleotide (dATP, dCTP, dGTP, and dTTP), 1X reaction buffer, 0.75 mM MgCl_2_, and 0.5 mM of each primer. The PCR consisted of an initial denaturation at 95 °C for 2 min and 40 cycles: 95 °C for 45 s, 55 °C for 45 s, and 72 °C for 45 s. A final extension step of 72 °C for 10 min was included. Amplification reactions were performed on a Veriti^TM^ 96-well Thermal Cycler (Thermo Fisher Scientific). PCR products were visualized on a 1.0% agarose gel as described above, sequenced (Macrogen, Seoul, Republic of Korea) using the primers 27F and 1492R, and manually edited using the ChromasPro software (http://technelysium.com.au/wp/chromaspro/ Accessed on 15 March 2022) to remove low-quality bases. Forward and reverse sequences were assembled using the Megamerger tool (http://www.bioinformatics.nl/cgi-bin/emboss/megamerger Accessed on 16 March 2022) to obtain sequences with lengths ranging in length from 1287 to 1498 bp. Partial sequences were compared to GenBank using BLAST 2.13.0 software [18]. 

### 2.4. Phylogenetic Analysis

A multiple sequence alignment of their 16S rRNA gene sequences was performed using MUSCLE [19] with default parameters to determine the phylogenetic relationships of the 57 bacterial isolates analyzed in this study. The alignment was trimmed using BMGE with the DNAPAM100 matrix to improve the accuracy of the phylogenetic inference [20]. A total of 84 16S rRNA gene sequences from closely related species were included in the alignment to provide a broader taxonomic context. The resulting alignment was used to infer a maximum likelihood (ML) phylogenetic tree that was inferred using RAxML v8.2.12 [21], conducting 20 ML tree searches under the standard GTRGAMMA model. The support values were calculated using the bootstrap procedure with 100 replicates to assess the reliability of the resulting tree. Finally, the phylogenetic tree was visualized using iTOL v6 [22].

### 2.5. Bacterial Characterization

#### 2.5.1. In Vitro Detection of Hydrolytic Activities

The hydrolytic activity of isolated bacteria was evaluated using five specific culture media and incubated for 24 to 48 h. The protease, lipase, chitinase, cellulase, and amylase enzymes were determined on an agar plate containing a specific substrate for each enzyme. All assays were performed in triplicate.

Protease activity was evaluated by growth on skim milk agar medium containing 28.0 g skim milk, 5.0 g casein hydrolysate, 2.5 g yeast extract, 1.0 g glucose, and 15.0 g agar per liter. Protein degradation is observed as a clear halo around the colony on a white background [23].

Lipase activity was determined by the method described by Slifkin [24]. Tween 80 agar contained the following per liter: 10.0 g peptone, 5.0 g NaCl, 0.1 g CaCl_2_, 5.0 mL Tween 80, and 15.0 g agar. A white halo around the microorganism indicates the hydrolysis of Tween 80, producing fatty acids that react with the calcium cations contained in the solid medium, forming a white precipitate, indicating a positive result.

Chitinase activity was evaluated in colloidal chitin medium according to the method described by Verma and Garg [25]. The medium contained the following per liter: 20.0 g colloidal shrimp chitin, 0.5 g yeast extract, 1.0 g MgSO_4_·7H_2_O, 1.36 g KH_2_PO_4_, and 15.0 g agar. After incubation, the chitinase activity was visualized as a halo of clearance around the colony on a white background.

Detection of cellulolytic activity was performed on agar plates containing per liter 1.0 g carboxymethylcellulose, 1.0 g peptone, 0.3 g urea, 2.0 g KH_2_PO_4_, 1.0 g (NH_4_)_2_SO_4_, 0.3 g CaCl_2_, 0.3 g/L MgSO_4_, 0.014 g/L ZnSO_4_, 0.002 g/L CoCl_2_, 0.05 g/L FeSO_4_, 0.016 g/L MnSO_4_, and 15.0 g agar. The cultures were incubated for five days at room temperature. After incubation, the plates were flooded with 0.1% Congo red solution for 20 min and then washed twice for 20 min with 1 M NaCl. The cellulolytic activity was observed as a transparent halo around the bacteria on a red background [26].

Amylase activity was evaluated on agar plates containing 2.0 g starch, 3.0 g beef extract, 6.0 g NaCl, and 12.0 g agar per liter. The plates were incubated at room temperature for one week and then flooded with a Lugol staining solution for 20 min. Amylase activity was observed as a clear halo around the microorganism on a dark background [26].

#### 2.5.2. Determination of Plant Growth-Promoting Traits

The colorimetric Salkowski’s method [27] was used to determine the production of Indole-3-acetic acid (IAA). After centrifugation, the bacterial supernatant was collected and mixed with Salkowski’s reagent (0.5 M FeCl_3_ in 35% HClO_4_) at a ratio of 1:1 (supernatant: Salkowski’s reagent). A color change from yellow to red indicated the presence of IAA.

The NFb semisolid medium [28] was used to assess the nitrogen-fixing ability of each bacterial isolate. This medium contained DL-malic acid (5.0 g/L), K_2_HPO_4_ (0.5 g/L), MgSO_4_·7H_2_O (0.2 g/L), NaCl (0.1 g/L), CaCl_2_·2H_2_O (0.02 g/L), agar (0.5 g/L), micronutrient solution (2 mL per liter), bromothymol blue solution (2 mL per liter of 0.5% in 0.2 N KOH), Fe (III)-EDTA (4 mL per liter of 1.64% *w*/*v*), and vitamin solution (1 mL per liter). The pH of the medium was adjusted to 6.8 with NaOH. The micronutrient solution contained CuSO_4_·5H_2_O (0.4 g/L), ZnSO_4_·7H_2_O (0.12 g/L), H_3_BO_3_ (1.4 g/L), Na_2_MoO_4_·2H_2_O (1.0 g/L), and MnSO_4_·H_2_O (1.5 g/L). The vitamin solution consisted of biotin (100 mg/L) and pyridoxal HCl (200 mg/L). Nitrogen fixation was evaluated by observing the development of a sub-surface whitish “veil-like” pellicle after incubation.

Solubilization of inorganic phosphate was evaluated using the Pikovskaya (PVK) solid medium [29]. The PVK medium contained glucose (10.0 g/L), yeast extract (0.5 g/L), (NH_4_)_2_SO_4_ (0.5 g/L), MgSO_4_·7H_2_O (0.1 g/L), Ca_3_(PO_4_)_2_ (5.0 g/L), KCl (0.2 g/L), MnSO_4_·2H_2_O (0.002 g/L), FeSO_4_·7H_2_O (0.002 g/L), and agar (15.0 g/L). The appearance of a clearing zone around bacterial colonies after incubation was used to determine the extent of phosphate solubilization.

Siderophore production was assessed using the Chrome Azurol S (CAS) method described by Schwynand and Neilands [30]. Bacterial supernatant (500 μL) was mixed with an equal volume of CAS solution, and the formation of an orange color from the initial blue indicated the production of siderophores.

All experiments were performed in triplicate.

#### 2.5.3. Evaluation of Growth Inhibition of Phytopathogenic Fungi In Vitro

An antagonism assay was performed using a 94 mm plate with 20 mL of PDA according to the procedure described by Sepúlveda-Chavera [31]. Fresh PDA plates were inoculated with agar disks from a one-week-old actively growing colony of the phytopathogenic fungi *Botrytis cinerea*, *Monilinia fructicola*, *Phytium* sp., *Geotrichum candidum*, *Fusarium oxysporum*, *Alternaria* sp., or *Macrophomina phaseolina* (obtained from the Microbial Culture Collection of the Universidad de Tarapacá) in the center of the plate. Additionally, 20 μL of each bacterial isolate was inoculated in 5 mm wells at a distance of 2.5 cm from the center of the plate. Plates only inoculated with fungi in the center were used as a control. Cultures were incubated at room temperature for 5–7 days, and a halo around the wells indicated the inhibition of fungal growth. All assays were performed in triplicate.

## 3. Results

### 3.1. Samples Characterization

The Jurasi Hot Springs are located at 4100 m.a.s.l. (18.2104° S, 69.5107° W; blue symbol, Figure 1) in the extreme north of the Atacama Desert (Arica and Parinacota Region, Chile). Table 1 shows the chemical analysis of the water and sediment samples obtained from Jurasi Hot Springs. The water sample had a neutral pH, temperatures ranging from 32 °C to 64 °C, and an electrical conductivity of 2.79 mS/cm. Higher levels of Ca^2+^ and Na^+^ cations were measured (188.65 mg/L and 158.26 mg/L, respectively), while Cl^−^ and SO_4_^2−^ were the most abundant anions (261.99 mg/L and 438.37 mg/L, respectively). The soil sample showed a pH value of 7.76 and an electrical conductivity of 4.38 mS/cm, with Ca^2+^ and Na^+^ as the predominant cations (400.95 mg/L and 304.63 mg/L, respectively) and Cl^−^ and SO_4_^2−^ as the prevailing anions (747.3 mg/L and 438.6 mg/L, respectively). B, Cu, Mn, Zn, Fe, and As were higher in the soil samples than in the water samples from Jurasi Hot Springs.

Amuyo Lagoons consist of three bodies of water situated at an elevation of 3700 m.a.s.l. (19.0581° S, 69.2528° W; red symbol, Figure 1), in the south of the Arica and Parinacota Region. Table 2 shows the chemical analysis of the water and sediment samples obtained from Amuyo Lagoons. The water samples from the three lagoons had neutral pH values and an electrical conductivity of 13.42 mS/cm, 14.39 mS/cm, and 13.63 mS/cm for Green, Red, and Yellow Lagoons, respectively, indicating the presence of saline waters. In all three locations, the cation Na^+^ was present at high levels (exceeding 2900 mg/L), with Ca^2+^ and K^+^ also present at levels exceeding 920 mg/L and 750 mg/L, respectively. The ion Cl^−^ was the most abundant anion, with a concentration of 4398.45 mg/L in Green Lagoon, 4920.30 mg/L in Red Lagoon, and 4622.10 mg/L in Yellow Lagoon. Moreover, the SO_4_^2−^ ion was the second most abundant anion present in the three samples (3046.39 mg/L in Green Lagoon, 2847.65 mg/L in Red Lagoon, and 2926.32 mg/L in Yellow Lagoon). These results suggest that the water of these three sites is saline with a significant contribution of sodium, chloride, and sulfate ions.

The sediment sample from Green Lagoon exhibited the highest electrical conductivity (23.09 mS/cm) and had a pH value of 7.91. An analysis of the cations indicated a predominance of Na^+^ ions (2329.90 mg/L), followed by Ca^2+^ (1547.63 mg/L) and K^+^ (836.12 mg/L). Similarly, an analysis of the anions showed a predominance of Cl^−^ (5550.19 mg/L), followed by SO_4_^2−^ (1954.32 mg/L). The microelements B, Cu, Mn, Zn, Fe, and As were detected in the sediment sample, with Fe being the most abundant element (197.27 mg/L).

For Red Lagoon, the sediment sample analysis revealed an electrical conductivity of 15.84 mS/cm and a pH of 7.91. Na^+^ was the predominant cation (2051.62 mg/L) followed by K^+^ (852.18 mg/L) and Ca^2+^ (735.38 mg/L). Chloride was the anion with the highest concentration (4546.65 mg/L), followed by SO_4_^2−^ (1096.21 mg/L). Moreover, the most abundant microelement was Fe (104.04 mg/L), followed by Mn (86.93 mg/L) and As (44.06 mg/L).

The sediment from Yellow Lagoon exhibited the lowest electrical conductivity (12.24 mS/cm) and the highest pH value of 8.21 of the three lagoons. Na^+^ was the prevalent cation (1566.87 mg/L), followed by Ca^2+^ (738.30 mg/L) and K^+^ (582.85 mg/L). Chloride and sulfate were the most abundant anions (3399.48 mg/L and 1159.09 mg/L, respectively). The most abundant microelement was Mn (74.56 mg/L), followed by Fe (49.45 mg/L) and As (48.21 mg/L).

The Polloquere Hot Springs are located at an elevation of 4300 m.a.s.l. (18.9128° S, 68.9987° W; gray symbol, Figure 1) in the southern part of the Arica and Parinacota Region. Table 3 shows the chemical analysis of the water and sediment samples obtained from Polloquere Hot Springs. The water sample had a pH value of 6.80 and an electrical conductivity of 8.55 mS/cm. The most abundant cation found was Na^+^ (1028.86 mg/L), followed by Ca^2+^ (610.24 mg/L). Minor contributions were made by K^+^ and Mg^2+^ (190.70 mg/L and 54.14 mg/L, respectively). Cl^−^ and SO_4_^2−^ anions were also detected in the Polloquere Hot Springs, and chloride was the most abundant (2236.86 mg/L). The sediment sample showed an electrical conductivity of 16.34, and the pH value was 6.35. The cations Na^+^ and Ca^2+^ were the most abundant ions (2167.51 mg/L and 1082.74 mg/L, respectively). Cl^−^ and SO_4_^2−^ were found to be the most abundant anions (4397.10 mg/L and 1602.57 mg/L, respectively). The prevalent microelements were Fe and Mn at concentrations of 126.32 mg/L and 104.06 mg/L, respectively, followed by As at 46.72 mg/L.

### 3.2. Bacterial Isolation, Identification, Distribution, and Functional Characterization

**Figure 2:**
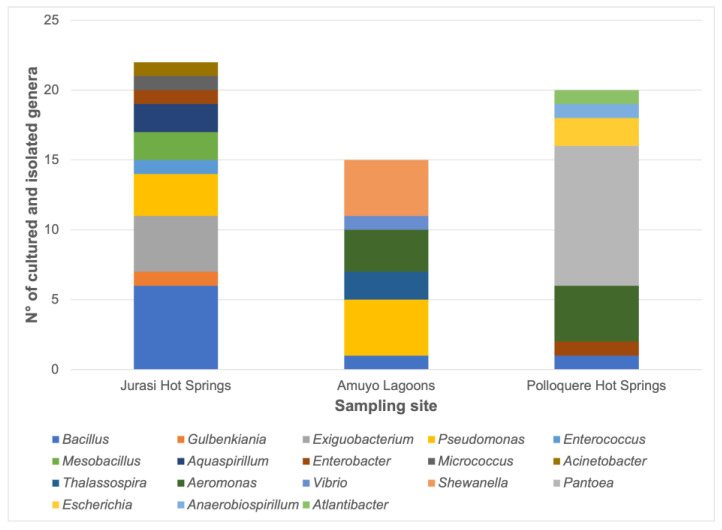
Number of cultured and isolated bacteria according to the sampling site. The quantity of each genus is represented as a color bar as is indicated in the image.

A total of 57 bacteria were isolated from three geothermal sites in the Arica and Parinacota Region, Chile (Figure 1). These included 22 strains from Jurasi Hot Springs, 15 isolates from Amuyo Lagoons, and 20 bacteria from Polloquere Hot Springs (Figure 2). Bacterial colonies were generally visible after 24–48 h of incubation at 25 °C. Most colonies were circular and had regular edges, with some showing pigmentation, including yellow and green fluorescent pigment production. The predominant microscopic morphology observed was rod-shaped bacteria with different lengths, whereas cocci were occasionally present.

Genomic DNA was extracted from the 57 isolates, and a ~1500 bp PCR product was amplified and sequenced to identify the microorganisms found in the samples from the three sites. A comparative sequence analysis of the isolates and bacterial 16S rRNA sequences available in GenBank (Table S1) revealed that the isolates belonging to *Pantoea* (10 isolates), *Bacillus* (8), *Pseudomonas* (7), and *Aeromonas* (7) were the most frequently isolated genera. Four isolates from the genera *Exiguobacterium* and *Shewanella* were obtained. One or two members from the following genera were isolated: *Gulbenkiania*, *Enterococcus*, *Mesobacillus*, *Aquaspirillum*, *Enterobacter*, *Micrococcus*, *Acinetobacter*, *Thalassospira*, *Vibrio*, *Escherichia*, *Anaerobiospirillum,* and *Atlantibacter* genera.

The frequency counts for the cultured bacteria, categorized by the genus and sampling site, are shown in Figure 2. The *Bacillus* genus appears to be the most frequently isolated from Jurasi Hot Springs, along with isolates belonging to *Gulbenkiania*, *Exiguobacterium*, *Pseudomonas*, *Enterococcus*, *Mesobacillus*, *Aquaspirillum*, *Enterobacter*, *Micrococcus,* and *Acinetobacter* that were also obtained. Members of the *Bacillus*, *Pseudomonas*, *Thalassospira*, *Aeromonas*, *Vibrio*, and *Shewanella* genera were isolated from Amuyo Lagoons. Meanwhile, the most frequently isolated bacteria from Polloquere Hot Springs belonged to the *Pantoea* genus, with additional members of *Bacillus*, *Enterobacter*, *Aeromonas*, *Escherichia*, *Anaerobiospirillum*, and *Atlantibacter* also obtained.

All the isolated bacteria were functionally characterized to determine their capability to produce hydrolases, plant growth-promoting traits, and the ability to inhibit phytopathogenic fungi (Table 4 and Table S1). The most abundant detected hydrolytic activity corresponded to proteolytic and lipolytic activities, with 35 and 31 isolates testing positive, respectively. Chitinolytic, cellulolytic, and amylolytic activities were also detected in 14, 21, and 24 isolates, respectively. Among the plant growth-promoting traits, phosphate solubilization, nitrogen fixation, auxin production, and siderophores production were exhibited in 43, 37, 34, and 11 isolates, respectively. Biocontrol activities against phytopathogenic fungi were less frequent, with 12 isolates capable of controlling *Alternaria* sp. and 10 bacteria capable of inhibiting *G. candidum*. Furthermore, the assays also showed significant inhibition of fungal growth against *B. cinerea* (seven), *F. oxysporum* (one), *Phytium* sp. (nine), *M. phaseolina* (eight), and *M. fructicola* (one).

### 3.3. Phylogenetic Analysis

**Figure 3:**
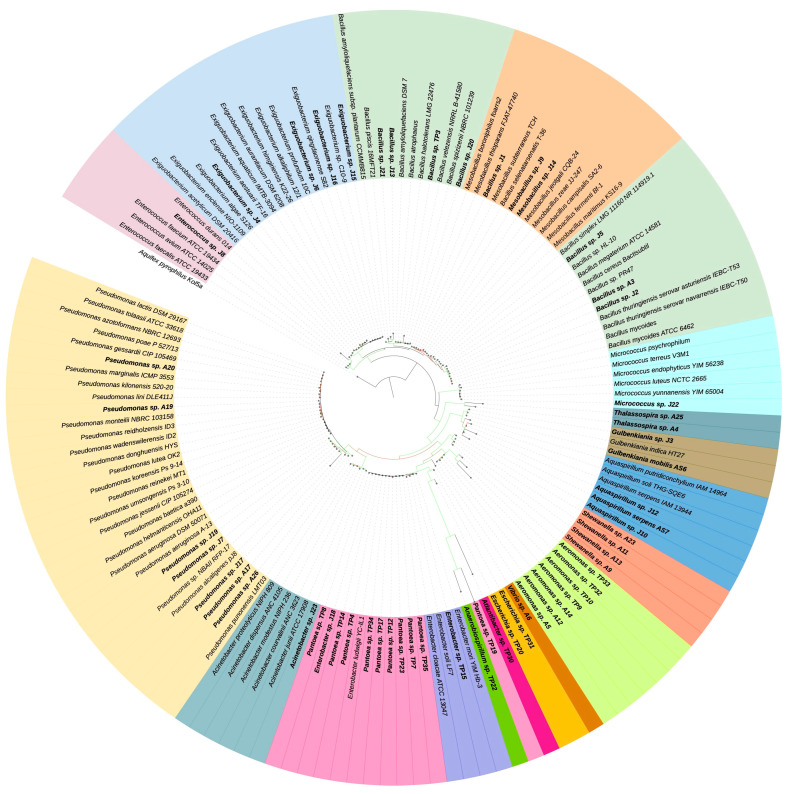
Phylogenetic tree based on the 16S rDNA gene sequences of the isolated bacteria and their closely related species obtained from GenBank. The tree was constructed using the maximum likelihood method. The tree presents the different genera highlighted in color.

**Figure 4:**
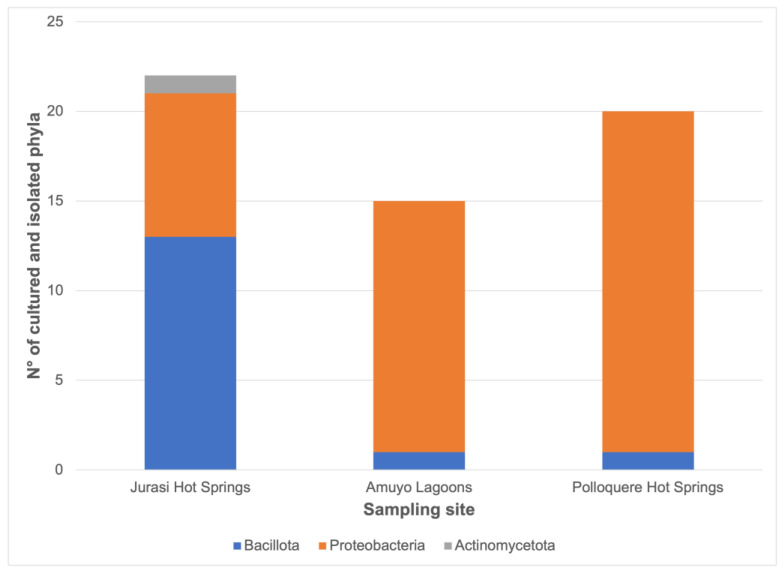
Number of cultured and isolated phyla according to the sampling site. The quantity of each genus is represented as a color bar, as indicated in the image.

The bacterial isolates underwent molecular analysis using the PCR-amplified product of the 16S rRNA gene (Figure 3). Based on the molecular characterization, the bacteria isolated from Jurasi Hot Springs were affiliated with three major phyla (Figure 4): 13 isolates belonging to the Bacillota phylum, 8 isolates from Proteobacteria, and 1 isolate from Actinomycetota phylum. From Amuyo Lagoons, 14 isolates belonged to the Proteobacteria phylum and 1 to the Bacillota phylum. Meanwhile, Polloquere Hot Springs yielded 19 isolates from the Proteobacteria phylum and 1 from the Bacillota phylum.

**Figure 5:**
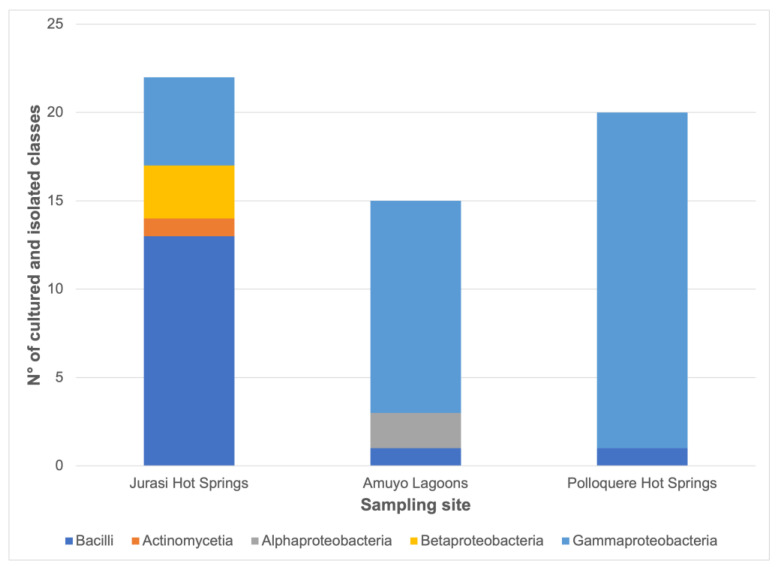
Number of cultured and isolated classes according to the sampling site. The quantity of each genus is represented as a color bar, as indicated in the image.

Figure 5 shows the number of cultured and isolated classes according to the sampling site. At Jurasi Hot Springs, the most common isolated class was Bacilli (13 isolates), followed by γ-Proteobacteria (5 isolates), β-Proteobacteria (3 isolates), and Actinomycetia (1 isolate). At Amuyo Lagoons, the most common class of isolated bacteria was γ-Proteobacteria (12 isolates), followed by α-Proteobacteria (2 isolates) and Bacilli (1 isolate). At Polloquere Hot Springs, the most common class was γ-Proteobacteria (19 isolates), with 1 isolate belonging to the Bacilli class.

## 4. Discussion

The Arica and Parinacota Region is located in the northernmost part of the Atacama Desert. It is characterized by extremely low annual rainfall along the coast, averaging only 1.6 mm per year. The region possesses limited humidity, resulting in significant temperature fluctuations between the day and the night. These conditions contribute to an arid environment with unfavorable soil composition and limited flora and fauna. Additionally, local water resources in this area are influenced by geothermal activity in the Andes Mountains, resulting in high concentrations of salts, boron, and arsenic, reducing the water quality suitable for agricultural activities [32]. Despite its limiting conditions, the Arica and Parinacota Region is mainly characterized by the production of different types of crops, including tomato, corn, alfalfa, oregano, and subtropical fruits, among others [33]. The agriculture developed in this region takes place in extreme conditions for crops, where high levels of salts and boron predominate. Under this scenario, the application of commercial biocontrollers or biostimulants shows erratic results [11], revealing the lack of functional bioproducts for this type of environment. In this sense, the bioprospection of saline-boric environments, such as Jurasi Hot Springs, Amuyo Lagoons, and Polloquere Hot Springs (Figure 1), could provide the opportunity to develop new bioproducts for environments that are considered extreme sites. The three sites have high contents of Na^+^, Ca^2+^, Cl^−^, and SO_4_^2−^ (Table 1, Table 2 and Table 3), showing EC in the water of 2.79 mS/cm for Jurasi Hot Springs, 13.42 mS/cm for Green Lagoon, 14.39 mS/cm for Red Lagoon, 13.63 mS/cm for Yellow Lagoon, and 8.55 mS/cm for Polloquere Hot Springs. Meanwhile, the EC for sediments was 4.38 mS/cm for Jurasi Hot Springs, 23.09 mS/cm for Green Lagoon, 15.84 mS/cm for Red Lagoon, 12.24 mS/cm for Yellow Lagoon, and 16.34 mS/cm for Polloquere Hot Springs, revealing that these sites have high saline conditions with an important contribution of Na^+^, Ca^2+^, Cl^−^, and SO_4_^2−^ ions. Moreover, the presence of boron in the sediments was determined in the three sites, reaching values of 20.38 mg/L for Jurasi Hot Springs, 29.46 mg/L for Green Lagoon, 18.22 mg/L for Red Lagoon, 30.03 mg/L for Yellow Lagoon, and 7.72 mg/L for Polloquere Hot Springs. Additionally, the presence of Cu was higher in the sediments of Amuyo Lagoons: 0.79 mg/L for Red Lagoon and 0.66 mg/L for the Green and Yellow lagoons. The concentration of Mn was higher in the sediments from Jurasi Hot Springs, reaching a value of 112.95 mg/L. Higher concentrations of Zn were measured in the Green (18.34 mg/L) and Red (14.06 mg/L) lagoons. Iron concentrations of 196.58 mg/L and 197.27 mg/L were the highest in the sediment samples from Jurasi Hot Springs and Green Lagoon, respectively. Meanwhile, the As concentrations were particularly higher in the sediment samples of Amuyo Lagoons (Green Lagoon: 42.60 mg/L; Red Lagoon: 44.06 mg/L; and Yellow Lagoon: 48.21 mg/L) and Polloquere Hot Springs (46.72 mg/L). These conditions represent a selective pressure exerted by abiotic factors on the microbial community, selecting microorganisms naturally adapted to their environments [34]. In this sense, it is possible to assume that bacteria isolated from Jurasi Hot Springs, Amuyo Lagoons, and Polloquere Hot Springs are naturally adapted to the saline-boric conditions imposed for these environments, allowing the development of new biobased products for the agriculture that is materialized under arid conditions.

In this study, a total of 57 bacterial isolates were obtained from Jurasi Hot Springs, Amuyo Lagoons, and Polloquere Hot Springs (Figure 2, Figure 3, Figure 4 and Figure 5, Table S1), including members of *Bacillus*, *Pseudomonas*, *Pantoea*, and *Exiguobacterium*, among others, which have been described as plant growth-promoting bacteria and biocontrollers of infectious plant diseases [35,36,37,38]. Isolates belonging to the *Bacillus* genus were present in the three geothermal sites, being the most abundant bacterium in Jurasi Hot Springs (Figure 2). Moreover, members of the *Pseudomonas* and *Shewanella* genera were frequently isolated from Amuyo Lagoons, and members of the genus *Pantoea* were cultivated many times from samples of Polloquere Hot Springs. These extreme-tolerant bacteria were isolated from three harsh environments, which encompass conditions, such as elevated temperatures; significant levels of electrical conductivity due to abundant salt content, including ions, such as Na^+^, Ca^2+^, Cl^−^, and SO_4_^2−^; and the presence of boron (as shown in Table 1, Table 2 and Table 3). Moreover, these bacteria showed the ability to degrade the soil organic matter (production of hydrolytic enzymes), the production of plant growth-promoting traits, and the biocontrol activity against phytopathogenic fungi (Table 4). Soil organic matter (SOM) refers to the material originally produced by plants or animals and subsequently returned to the soil. It undergoes degradation and plays a pivotal role in establishing soil quality and fertility. This degradation process is facilitated by external enzymes, including extracellular microbial hydrolases, which break down large molecules into soluble components that plants can assimilate [39]. According to our analysis, protease and lipase activity were the most frequently detected hydrolases (61.4% and 54.4%, respectively). Hydrolases are among the most studied microbial biocatalysts in soils, constituting a category of enzymes that facilitate the hydrolysis of covalent bonds and have significant industrial applications. Microorganisms use hydrolases to break down natural organic polymers as an energy source. They also play a role in metabolizing xenobiotic compounds, such as pesticides [40]. Novel microbial hydrolases with promising biotechnological potential, such as lipases and proteases, would represent a source of novel enzymes that could be explored to develop new tools for agriculture to improve soil quality by increasing fertility and reducing the use of chemical pesticides.

A limited amount of research is available on bacteria isolated from regions characterized by extreme conditions and exhibiting PGP activity. These traits have been evaluated under different scenarios involving factors, such as salinity, temperature, and, in certain instances, pH. Among the plant growth-promoting traits detected in isolated bacteria from Jurasi Hot Springs, Amuyo Lagoons, and Polloquere Hot Springs, phosphate solubilization (75.4%), nitrogen fixation (64.9%), and auxin production (59.6%) were the PGP activities with higher relative frequencies (Table 4). In the study by Tuesta-Popolizio et al. [15], strains isolated from the Los Negritos geothermal site showed various activities related to plant growth promotion, including auxin production, solubilization of inorganic phosphate, and siderophores formation. Amaresan et al. [41] isolated a total of 102 bacteria from the active volcano Barren Island, India. The results obtained through 16S rRNA gene sequencing revealed that the plant growth-promoting (PGP) bacteria belonged to a diverse range of 22 species belonging to 13 genera, including members of *Bacillus*, *Escherichia*, *Ralstonia*, *Enterobacter*, and *Staphylococcus* genera. These bacteria were capable of producing auxins (57.8%), siderophores (55.9%), and solubilizing phosphate (33.3%). In the research conducted by Gaete et al. [14], a total of 71 bacterial strains were isolated from two different locations: Coppermine Peninsula in Antarctica and Lejia Lake in the Atacama Desert. These isolated bacterial strains displayed in vitro traits associated with plant growth-promoting activities, belonging to four phyla (Proteobacteria, Actinobacteria, Firmicutes, and Bacteroidetes), with *Pseudomonas* being the most represented genus in both sites. These studies suggest the possibility of obtaining extremotolerant bacteria from extreme habitats to generate new biobased products for agriculture developed in different environments.

Regarding the in vitro antagonistic activity of isolated bacteria against phytopathogenic fungi, a low relative frequency of antifungal activity was detected in the isolated strains (Table 4 and Table S1), ranging from 1.8% (*F. oxysporum* and *M. fructicola*) to 21.1% (*Alternaria* sp.). Similar results were obtained by Amaresan et al. [41], who observed a frequency of biofungicide activity of 29.9% against *Macrophomina* sp., 19.6% against *Rhizoctonia solani*, and 14.7% against *Sclerotium rolfsii*. The biocontrol mechanisms employed by plant-associated bacteria to suppress pathogens encompass a range of strategies, including competing for resources, like iron, nutrients, and space. These mechanisms also involve the production of antibiotics and lytic enzymes [42]. Lytic enzymes, particularly extracellular proteases, have been explored for their biocontrol capabilities against multiple plant pathogens [43]. For instance, a strain of *Bacillus amyloliquefaciens* isolated from the rhizosphere of Jute displayed biocontrol activity against *Macrophomina phaseolina*, *F. oxysporum*, *Fusarium semitectum*, and *Alternaria alternata* through protease-mediated mechanisms [44]. In this study, further investigation is required to unveil the precise mechanisms by which bacteria sourced from three geothermal sites from the Arica and Parinacota Region inhibit the growth of phytopathogenic fungi.

Our findings underscore the potential for creating novel biobased agricultural products developed for arid and semi-arid environments. Arid and semi-arid habitats are prevalent across the globe, covering approximately 30% to 40% of the Earth’s land surface [8]. In the north of the Atacama Desert, coastal regions experience minimal annual rainfall, averaging around 1.6 mm per year [45]. The scarcity of moisture leads to pronounced temperature fluctuations between the day and the night, exacerbating the arid conditions; this, in turn, contributes to impoverished soil quality and a diminished presence of plant and animal life. Additionally, geothermal activities in the Andes Mountains significantly affect water resources in this region, causing high concentrations of salts, boron, and arsenic. Consequently, the water quality available for agricultural purposes is compromised, presenting significant challenges alongside other specific limitations [32]. These constraints limit not only agricultural production but also the activity of commercial biostimulants and biocontrollers, whose functionality is limited by the presence of salts and boron [11]. Additionally, some studies [46,47,48] have proposed the application of PGP bacteria isolated from extreme environments as inoculants in comparable conditions to those in which they were originally found. Consequently, bacteria obtained from a cold desert could be applied to mitigate the effects of cold stress. In contrast, bacteria isolated in arid regions could provide a solution to mitigate the effects of drought.

## 5. Conclusions

Jurasi Hot Springs, Amuyo Lagoons, and Polloquere Hot Springs, located in the extreme northern region of the Atacama Desert within the Arica and Parinacota Region of Chile, host a wide array of bacteria. These microorganisms showed interesting plant growth-promoting traits, including phosphate solubilization, nitrogen fixation, and auxin and siderophores production, which could stimulate plant growth. Moreover, the presence of hydrolytic enzymes was detected, indicating the ability of these bacteria to degrade organic matter in soil, making available nutrients for plants. Furthermore, antifungal activity was detected in low frequencies, showing the possibility of developing new biocontrollers for phytopathogenic fungi.

Based on the molecular characterization of culturable bacteria, three major phyla were detected in Jurasi Hot Springs (Bacillota, Proteobacteria, and Actinomycetota phylum); two phyla were isolated from Amuyo Lagoons (Proteobacteria and Bacillota); and two phyla were obtained from Polloquere Hot Springs (Proteobacteria and Bacillota). These phyla exhibit plant growth-promoting characteristics and could potentially enhance plants’ resistance to salinity and boron stress. These bacteria hold significant promise for developing novel biobased agricultural products for arid and semi-arid regions, and they can also be harnessed for various biotechnological applications.

## 6. Patents

A patent application was requested to INAPI under the application code PCT/CL2022/050102.

## Figures and Tables

**Table 1:** Physicochemical parameters of Jurasi Hot Springs.

Parameter	Jurasi Hot Springs	Jurasi Hot Springs
Altitude (m.a.s.l.)	4100	4100
Type of Sample	water	sediment
Temperature (°C)	32–64	35–65
Electrical Conductivity (mS/cm)	2.79 ± 0.21	4.38 ± 0.27
pH	6.97 ± 0.09	7.76 ± 0.11
Ca (mg/L)	188.65 ± 17.5	400.95 ± 23.3
Mg (mg/L)	3.47 ± 0.22	10.80 ± 0.37
K (mg/L)	26.15 ± 0.16	87.35 ± 0.27
Na (mg/L)	158.26 ± 1.54	304.63 ± 3.33
Cl^−^ (mg/L)	261.99 ± 5.48	747.30 ± 9.98
SO_4_^2−^ (mg/L)	438.37 ± 5.40	438.60 ± 8.53
NO_3_^−^ (mg/L)	0.00 ± 0.00	3.80 ± 0.28
HCO_3_^−^ (mg/L)	69.54 ± 3.22	69.54 ± 4.51
CO_3_^2−^ (mg/L)	0.00 ± 0.00	0.00 ± 0.00
H_2_PO_4_^−^ (mg/L)	1.50 ± 0.07	74.65 ± 2.97
B (mg/L)	7.30 ± 0.87	20.38 ± 2.05
Cu (mg/L)	0.02 ± 0.00	12.38 ± 0.21
Mn (mg/L)	0.68 ± 0.05	112.95 ± 1.99
Zn (mg/L)	0.26 ± 0.09	7.01 ± 1.26
Fe (mg/L)	1.47 ± 0.10	196.59 ± 3.27
As (mg/L)	0.19 ± 0.05	15.08 ± 0.89

**Table 2:** Physicochemical parameters of Amuyo Lagoons.

Parameter	Green Lagoon	Green Lagoon	Red Lagoon	Red Lagoon	Yellow Lagoon	Yellow Lagoon
Altitude (m.a.s.l.)	3700	3700	3700	3700	3700	3700
Type of Sample	water	sediment	water	sediment	water	sediment
Temperature (°C)	21–35	21–35	22–25	22–25	22–52	22–52
Electrical Conductivity (mS/cm)	13.42 ± 0.31	23.09 ± 0.46	14.39 ± 0.33	15.84 ± 0.23	13.63 ± 0.28	12.24 ± 0.32
pH	7.16 ± 0.12	7.91 ± 0.06	7.59 ± 0.12	7.91 ± 0.17	7.01 ± 0.08	8.21 ± 0.11
Ca (mg/L)	926.67 ± 23.77	1547.63 ± 58.30	970.50 ± 33.87	735.38 ± 48.22	936.33 ± 43.78	738.30 ± 42.90
Mg (mg/L)	58.96 ± 3.57	74.08 ± 6.76	65.27 ± 5.67	33.72 ± 3.27	61.23 ± 6.31	31.48 ± 4.27
K (mg/L)	759.98 ± 10.98	836.12 ± 22.10	841.47 ± 31.03	852.18 ± 29.87	779.49 ± 41.31	582.85 ± 24.84
Na (mg/L)	2960.03 ± 58.66	2329.90 ± 68.70	3079.33 ± 71.52	2051.62 ± 65.03	2999.77 ± 67.85	1566.87 ± 58.80
Cl^−^ (mg/L)	4398.45 ± 66.43	5550.19 ± 76.65	4920.30 ± 55.98	4546.65 ± 56.87	4622.10 ± 79.65	3399.48 ± 48.68
SO_4_^2−^ (mg/L)	3046.39 ± 43.65	1954.32 ± 52.01	2847.65 ± 55.65	1096.21 ± 37.86	2926.32 ± 66.50	1159.09 ± 36.87
NO_3_^−^ (mg/L)	0.00 ± 0.00	3.80 ± 0.63	1.90 ± 0.45	0.38 ± 0.13	0.00 ± 0.00	0.00 ± 0.00
HCO_3_^−^ (mg/L)	727.93 ± 23.40	799.40 ± 32.43	687.27 ± 43.17	191.24 ± 23.41	671.00 ± 45.21	185.44 ± 42.11
CO_3_^2−^ (mg/L)	0.00 ± 0.00	82.80 ± 2.80	0.00 ± 0.00	0.00 ± 0.00	0.00 ± 0.00	22.70 ± 0.56
H_2_PO_4_^−^ (mg/L)	8.99 ± 0.23	11.76 ± 0.43	12.52 ± 0.35	69.96 ± 1.02	9.54 ± 0.55	5.07 ± 0.35
B (mg/L)	27.10 ± 0.11	29.46 ± 0.16	28.73 ± 0.22	18.22 ± 0.34	27.14 ± 0.43	30.03 ± 0.49
Cu (mg/L)	0.04 ± 0.01	0.66 ± 0.10	0.03 ± 0.01	0.79 ± 0.12	0.04 ± 0.01	0.66 ± 0.14
Mn (mg/L)	4.70 ± 0.56	63.65 ± 3.65	2.94 ± 0.35	86.93 ± 5.45	3.36 ± 0.54	74.56 ± 3.68
Zn (mg/L)	0.59 ± 0.02	18.34 ± 0.32	0.77 ± 0.03	14.06 ± 0.43	0.52 ± 0.02	3.76 ± 0.66
Fe (mg/L)	4.94 ± 0.22	197.27 ± 2.57	7.97 ± 0.44	104.04 ± 3.84	6.19 ± 0.53	49.45 ± 4.88
As (mg/L)	18.08 ± 0.76	42.60 ± 2.56	22.24 ± 1.05	44.06 ± 3.48	16.07 ± 0.65	48.21 ± 4.03

**Table 3:** Physicochemical parameters of Polloquere Hot Springs.

Parameter	Polloquere Hot Springs	Polloquere Hot Springs
Altitude (m.a.s.l.)	4300	4300
Type of Sample	water	sediment
Temperature (°C)	40–75	40–75
Electrical Conductivity (mS/cm)	8.55 ± 0.33	16.34 ± 0.53
pH	6.80 ± 0.15	6.35 ± 0.09
Ca (mg/L)	610.24 ± 10.50	1082.74 ± 22.47
Mg (mg/L)	54.14 ± 4.12	89.80 ± 7.77
K (mg/L)	190.70 ± 5.06	265.52 ± 9.93
Na (mg/L)	1028.86 ± 23.55	2167.51 ± 54.67
Cl^−^ (mg/L)	2236.86 ± 33.99	4397.10 ± 76.85
SO_4_^2−^ (mg/L)	808.12 ± 12.76	1602.57 ± 22.32
NO_3_^−^ (mg/L)	0.00 ± 0.00	0.00 ± 0.00
HCO_3_^−^ (mg/L)	0.00 ± 0.00	0.00 ± 0.00
CO_3_^2−^ (mg/L)	0.00 ± 0.00	0.00 ± 0.00
H_2_PO_4_^−^ (mg/L)	0.00 ± 0.00	0.00 ± 0.00
B (mg/L)	5.75 ± 0.66	7.72 ± 0.65
Cu (mg/L)	0.44 ± 0.03	0.57 ± 0.02
Mn (mg/L)	69.12 ± 1.13	104.06 ± 3.09
Zn (mg/L)	9.02 ± 0.54	10.33 ± 0.63
Fe (mg/L)	110.22 ± 3.33	126.32 ± 4.27
As (mg/L)	22.36 ± 0.97	46.72 ± 1.38

**Table 4:** Functional characterization of bacterial cultures isolated from three geothermal sites from the Arica and Parinacota Region, Chile.

Activity	N° Isolates with Positive Reaction	Relative Frequency (%)
Protease	35	61.4
Lipase	31	54.4
Chitinase	14	24.6
Cellulase	21	36.8
Amylase	24	42.1
Auxin production	34	59.6
Siderophores production	11	19.3
N_2_ fixation	37	64.9
Phosphate solubilization	43	75.4
*Botrytis cinerea* *	7	12.3
*Fusarium oxysporum* *	1	1.8
*Alternaria* sp. *	12	21.1
*Geotrichum candidum* *	10	17.5
*Phytium* sp. *	9	15.8
*Macrophomina phaseolina* *	8	14.0
*Monilinia fructicola* *	1	1.8

* Biocontrol activity against phytopathogenic fungus.

## Data Availability

Data are contained within the article or Supplementary Materials.

## Supplementary Materials

The following supporting information can be downloaded at https://www.mdpi.com/article/10.3390/microorganisms11112635/s1. Table S1: Identification and biochemical characterization of isolated bacteria.
